# *Magnaporthiopsis maydis* reduces growth in maize plantlets without affecting root resistance to water flow

**DOI:** 10.3389/fpls.2026.1851534

**Published:** 2026-06-03

**Authors:** Leire Molinero-Ruiz, Ana B. García-Carneros, Luca Testi

**Affiliations:** 1 Crop Protection Department, Institute for Sustainable Agriculture, Spanish National Research Council, Cordoba, Spain; 2 Agronomy Department, Institute for Sustainable Agriculture, Spanish National Research Council, Cordoba, Spain

**Keywords:** *Cephalosporium maydis*, endophyte, *Harpophora maydis*, root physiology, vascular infection, *Zea mays* L.

## Abstract

Late wilt is a disease caused by the soil-borne fungus *Magnaporthiopsis maydis* that severely limits the production of maize in countries with warm to hot climates. A particular characteristic of this disease is that, although the pathogen penetrates the roots in the first weeks after sowing, the symptoms appear suddenly around flowering. Additionally, even with air temperatures and relative humidity that are optimal for disease development during the crop cycle, adequate soil water content results in undetected disease. Overall, physiological alterations caused by *M. maydis* in maize are poorly understood. The objectives of this work were to analyse how maize roots and their hydraulic resistance are affected by *M. maydis*, and describe alterations in maize organs development at early growth stages. An experiment was conducted under greenhouse conditions for 6–8 weeks in 2018, 2020, and 2022. When dry weights of roots and aboveground parts were recorded, the fungus caused similarly stunted growth of both types of organ. Second, the fungus reduced the growth of any root diameter in an equal measure. With regard to root resistance measurements, unaltered - or in some instances increased - water flow was recorded in infected roots. This suggests that decreased radial resistance upon infection could mask high xylem resistance associated with vascular infection, thus resulting in a decreased resistance of the roots. A latent lifestyle of *M. maydis* would explain its internal growth in maize without apparently affecting water transport from the roots to the vascular system for some time (latent period), with the wilt appearing suddenly and at around plant flowering time (pathogen period).

## 1 Introduction

Late wilt, caused by the soil-borne fungus *Magnaporthiopsis maydis* (synonym *Cephalosporium maydis*) (Samra et al., 1963) greatly limits the production of maize in countries with warm to hot climates, like Egypt, India, Portugal, Spain and Turkey (El-Shafey and Claflin, 1999; Molinero-Ruiz et al., 2010; Yuceer et al., 2025). The pathogen penetrates the roots of plantlets in the first weeks after sowing (Sabet et al., 1970), but wilt symptoms do not appear until around flowering, with leaves drying up and steadily becoming brittle up to plant maturity (Molinero-Ruiz et al., 2010). Maize late wilt is mainly controlled by means of genetic resistance, an option that is limited by the existence of a diversity of aggressive strains within *M. maydis* populations (Ortiz-Bustos et al., 2016; Zeller et al., 2002), and, in some cases, by a partial expression of resistance, which greatly depends on environmental conditions (Ortiz-Bustos et al., 2016). In fact, the environment, as well as particular crop practices, play major roles in the expression of late wilt. Options like the sowing date selection, or water availability (Bergstrom et al., 2008) have been studied to reduce late wilt severity and losses. Frequent watering or saturated soils have been associated with reduced late wilt (Samra et al., 1966), and moisture stress is reported to be a major predisposing factor for the disease (Abd El-Rahim et al., 1998). Moving from sprinkler to drip, consequently increasing the water supply and the volume of root system wetted under high frequency irrigation, reduced severity of late wilt (Degani et al., 2018). Moreover, it was shown that even under optimal conditions for disease across the crop cycle (air temperature 26-28°C and relative humidity 43-45%), a good soil water content results in undetected disease, although reductions in yield and aboveground biomass can still occur (Ortiz-Bustos et al., 2019). Apart from the great influence of water regimes on growth and production variables of infected maize when air temperature and humidity are favourable for late wilt development, it remains to be seen to what extent wilt symptoms, which appear subsequently to root infection by *M. maydis*, are caused by the restriction of xylem water flow imposed by the pathogen inside maize tissues. Actually, previous works suggest that the root system may well be the first and most important target organ for *M. maydis* (Ortiz-Bustos et al., 2019). Infection of the maize root system by the pathogen early in the season not only results in late wilt symptoms around flowering time and yield reduction, but also seems to alter root functioning throughout the crop season well before any symptom appears (Ortiz-Bustos et al., 2019).

Maize late wilt visual symptoms are somewhat similar to those induced by generic water deficit (leaf tissue changing first to a pale green colour, then the whole leaf rolling inwardly lengthwise, and an eventual drying up of leaves, that become brittle). Symptoms are magnified by water stress (Abd El-Rahim et al., 1998; Degani et al., 2018), they appear late in the season, when atmospheric water demand is high (Ortiz-Bustos et al., 2019), and the global geographical distribution of the yield reduction is heavily correlated with warm and dry climates, with high transpiration rates (Ortiz-Bustos et al., 2019). Last, as a countercheck, it would seem that, when any “exogenous” water stress is purposely avoided by keeping the soil close to field capacity during the whole season, the visual symptoms are feeble or may not appear at all (Ortiz-Bustos et al., 2019). All these findings lead to the hypothesis that the primal effect of *M. maydis* on the plant is an increase in its resistance to water flow.

The Soil-Plant-Atmosphere-Continuum modelling paradigm (Williams et al., 2001) describes the water flux through the plant as directly proportional to the difference between the water potentials in two different points of the continuum and inversely proportional to the sum of the hydraulic resistances exerted by the medium through which the water flows. García-Tejera et al. (2017) define the flow through the root system (equal, at equilibrium to the transpiration T) according to Equation 1:

(1) $$T=\left(\Psi_{s}-\Psi_{c}\right)/\left(R_{s}+R_{root}\right)$$

Where Ψ_s_ and Ψ_c_ are the water potentials of the soil and the plant collar respectively, and R_s_ is the resistance from the general soil to the root surface (function of the soil type and water content). The hydraulic resistance of the root (R_root_) is:

(2) $$R_{root}=R_{r}+R_{x}$$

where R_r_ is the radial resistance, or the resistance exerted on water flowing from the root surface to the inner xylem vessels, i.e. perpendicularly to the root axis; and (R_x_) is the resistance for water flowing axially inside the root xylem vessels towards the stem. A pathogen increasing root resistance must therefore augment one of these two components, or both.

Physiological alterations caused by *M. maydis* in maize are poorly understood. In this study, we explored whether the leaf wilting and yield reduction are direct effects of the pathogen on the aboveground organs, or are caused by root system impairment. The objectives were to: a) analyse how maize root characteristics are affected by *M. maydis* at early growth stages; b) determine whether *M. maydis* increases root resistance at initial stages after infection; and c) describe the alteration in maize root development caused by the pathogen at early growth stages.

## 2 Materials and methods

### 2.1 Inoculation and growth of plants

An experiment was conducted under greenhouse conditions following the methodology by Tej et al. (2018) for inoculation and plant growth. A monoconidial culture of isolate Hm01–15, obtained from a symptomatic maize plant collected in Almodovar del Rio (Córdoba, Spain) in the spring of 2015 and used in previous works by our group (Tej et al., 2018), was transferred to PDAs and incubated at 28 °C with a 12-h photoperiod. After 10 days, fungal conidial suspensions were prepared by flooding the plates with sterile deionized water, scraping and filtration of the suspensions through four layers of sterile cheesecloth. Conidial concentration was adjusted to 10^6^ conidia mL^−1^ using a Neubauer hemacytometer. Soil for growing the plants was prepared as a sterilized mixture sand:silt:cornflour (5:2:1, vol:vol:vol) to which the conidial suspension was added at a final concentration of 10^4^ conidia g mixture^−1^ prior to incubation at 22–24 °C in the dark for 3 weeks. The same volume of deionized water was added to the mixture of the control treatment.

Seeds of the maize variety MO1501 (Monsanto Madrid, Spain), genetically susceptible to late wilt (Tej et al., 2018), were surface-sterilized and incubated in the dark until radicles were 5–10 mm long. Then, the (infested or control) soil mixture was added to 0.3-L pots to which individual seedlings were transplanted 24 h later. Plants were grown for 6 weeks in the greenhouse at 25 ± 2 °C and a 12-h photoperiod per day. They were fertilized three times a week with 30 mL of Hoagland’s nutrient solution per pot, and watered as required. At the end of the experiment, the height and weight of the roots and aboveground parts (stem and leaves) of each plant were recorded. The experiment was set up as a completely randomised design with ten replications (individual plants) from April 27 to June 7, 2018. The experiment was repeated from April 23 to June 11, 2020 and from March 31 to May 23, 2022, under the same greenhouse conditions. Twenty replications were established in 2022, since ten of them were used for destructive handling and measurements of plants.

### 2.2 Measurements of plant traits upon infection, and statistical analysis

The experiment was finalised and plants were uprooted when they were at the V3-V4 growth stages (Abendroth et al., 2011). Therefore, the variables evaluated were those of vegetative growth traits. Dry weights of roots and aboveground parts (g) were recorded at the end of each experiment. For the determination of dry weights, plant tissues were kept in a stove at 80°C for 48 h. Leaf area was measured in 2020 and 2022, using a flatbed scanner (HP Scanjet G3110). Height and stem diameter of each plant were noted in 2022.

In order to analyse the root system, the plants (four replications of each treatment) were uprooted and their roots cleaned and washed at final time. Thereafter, they were kept in water:ethanol (50:50, vol:vol) for scanner analysis. Roots were prepared in a tray and scanned with a flatbed scanner (HP Scanjet G3110). The images were processed and analysed with the root image analysis software Rhizovision Explorer (version 2.0.3, Seethepalli et al., 2021) to obtain root length and surface distributions.

**Figure 1:**
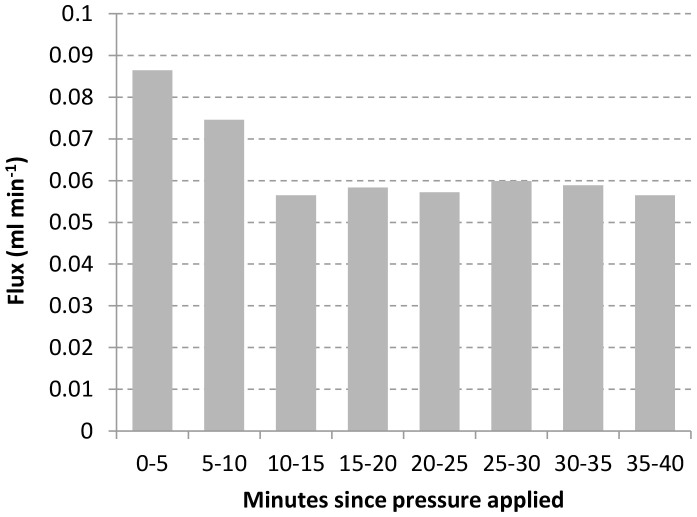
Time span of the water flux through the pressurized root system of a young maize plant (control) at a constant pressure of 0.4 MPa and temperature of 25 °C.

Root resistance (R_root_) was determined for ten replications of each control and inoculated treatments in 2022 by separating the whole plant root system at the collar, submerging it in water in a beaker. The beaker with the intact root system was inserted in a Scholander pressure chamber (model 3000F0, Soilmoisture Equipment Corporation, Santa Barbara, CA, USA) and closed around the collar. The water efflux from the collar when the root system was kept under a constant pressure of 0.4 MPa was collected by imbibition of small plastic capsules filled with cotton, previously weighed individually on a precision laboratory scale (Mettler Toledo, Greifensee, Switzerland). The capsules were replaced at every 5 minutes of imbibition with the 5-min flow data being obtained from the difference in the mass of each capsule. When the pressure and temperature were kept stable, the flow tended to stabilize after 10–15 minutes (**Figure 1**), and the average flow of the following 30–40 minutes together with the root system surface was taken for the calculation of R_root_. More details about the methodology are given in Garcia-Tejera et al. (2016).

All the variables were analysed using Welch’s t-test, after log transformation for the ratios, because it provides a more robust framework for small sample sizes (our case), where testing for the homogeneity of variance (e.g., via Levene’s test) often lacks sufficient statistical power. The significance level α = 0.05 was considered.

### 2.3 Microbiological and molecular diagnosis of *Magnaporthiopsis maydis* in the inoculated plants

The presence of *M. maydis* in inoculated plants was confirmed by means of microbiological and molecular analyses of samples from six replications, as described in Ortiz-Bustos et al. (2019). At the end of the experiment, cross sections from the first internode of the stem (3–5 cm aboveground) of each inoculated and control plant were sampled and stored at room temperature for one month. Each stem section was divided into two to six pieces that were surface-disinfested for 3 min by immersion in 20% household bleach (40 g of active chlorine per litre), rinsed in deionized water for 3 min, and air dried. Segments 2 to 4 mm long of maize tissue were aseptically transferred to petri plates containing PDA and incubated at 25 °C for 72 h in darkness. Colonies were morphologically confirmed by observation under the stereomicroscope.

For molecular diagnosis, total genomic DNA from dry stem tissues of inoculated and control plants was individually purified using the i-genomic Plant DNA Extraction Minikit (iNtRON Biotechnology, Sangdaewon-Dong, Korea) following the manufacturer’s instructions. DNA sample quality and concentration were determined with a QubitTM 3.0 Fluorometer (InvitrogenTM, Carlsbad CA, USA), and they were adjusted to a final concentration of 10 ng/μL and stored at –20 °C until required. Primers used were A200a (forward primer, 5’-CCGACGCCTAAAATACAGGA-3’) and A200b (reverse primer: 5’-GGGCTTTTTAGGGCCTTTTT-3’) (Drori et al., 2013). Optimized PCR assays were carried out in a final volume of 25 mL containing 0.4 µM each primer, 800 µM dNTPs, 2.5 mL 10x PCR buffer (800 mM tris–HCl, pH 8.3–8.4 at 25°C, 0.2% Tween 20 wt/V), 0.15 U Horse-Power-Taq DNA polymerase (Canvax, Córdoba, Spain), 2.5 mM MgCl_2_ and 20 ng of fungal DNA. The following profile was set for amplifications: 2 min initial denaturation at 94 °C; 35 cycles of 30 s denaturation at 94 °C, 30 s annealing at 55 °C, and 1 min of extension at 72 °C, and a final extension step of 5 min at 72 °C. Mycelial DNA of the fungus grown onto PDAs and water were used as positive and negative controls, respectively, of amplification. All reactions were done in a T1 Thermocycler (Whatman Biometra, Göttingen, Germany). Amplification products were separated by horizontal electrophoresis in 2% agarose gels containing 0.05 µl/ml GoldView Nucleic Acid Stain (Biotech, Beijing, China), and visualized over a UV light source. A 100- to 1,000-bp ladder (Canvax Biotech SL, Cordoba, Spain) was included in the electrophoresis.

## 3 Results

### 3.1 Aerial and root systems of maize infected by *Magnaporthiopsis maydis*

The main biometrics of infected and healthy young maize plants for the three experimental replicates are summarized in **Table 1**. The experimental conditions were slightly different due to the inter-annual variations and different plant ages at the end of the experiment; for example, the 2018 and 2020 plants were 6 and 7 weeks old, respectively, and the 2022 plants were uprooted when they were 8 weeks old. Nevertheless, control and inoculated plants were subjected to exactly the same conditions in each experiment.

**Table 1:** Averages and standard deviation of biometrics taken on the experimental plants at the end of the experiments.

Experiment	Treatment		Height (cm)	Stem diam. (mm)	Leaf area (cm^2^)	Specific leaf area (cm^2^/g)	Root dry mass (g)	Above-ground dry mass (g)	Total root length (cm)	Total root surface (cm^2^)	Roots/above-ground mass	Roots/leaves surface
2018	Control	Average					0.44	0.73	2006.6	340.9	0.61	
		StdDev					0.1	0.19	527.8	86.0	0.09	
	Inoculated	Average					0.26	0.41	1379.1	239.9	0.64	
		StdDev					0.05	0.09	182.5	30.0	0.11	
		Different					*	*	*	*		
2020	Control	Average			92.9	198.9	0.42	0.49	911.7	117.1	0.88	1.25
		StdDev			16.3	32.2	0.1	0.13	200.1	30.6	0.14	0.14
	Inoculated	Average			54.5	203.8	0.23	0.28	550.7	72.3	0.82	1.35
		StdDev			15.4	43.2	0.08	0.08	223.6	22.2	0.15	0.3
		Different			*		*	*	*	*		
2022	Control	Average	68.4	6.13	367.1	380.1	0.92	1.56	5915.4	905.4	0.64	2.59
		StdDev	5.1	0.59	95.9	59.7	0.16	0.48	898.3	125.2	0.19	0.55
	Inoculated	Average	35.2	3.61	142.7	524.3	0.39	0.45	2416.6	354.8	0.93	2.52
		StdDev	4.5	0.58	44.1	51.3	0.12	0.16	828.7	132.1	0.27	0.53
		Different	*	*	*	*		*	*	*	*	

Root length and surface refer to absorbent roots (Ø< 2mm). Asterisks denote significant differences (Welch T-test, α=0.05).

A noticeably harmful impact of *M. maydis* on several growth variables (height and stem diameter) was observed during the experiment conducted in 2022 (**Table 1**). Specifically, the inoculated plants exhibited a significantly (*P*< 0.0001) reduced height compared to the control group, measuring 35.2 cm versus 68.4 cm, respectively (48%). Furthermore, *M. maydis* led to a notable (41%) decrease in stem diameter, with measurements of 3.61 mm for the inoculated treatments and 6.13 mm for the controls.

Leaf area was significantly lower in inoculated plants in both 2020 and 2022 experiments (**Table 1**). The fungus reduced plant leaf area by about 41% in 2020 (plants with 3 leaves) and 61% in 2022 (plants with 6–7 leaves). Other evidence of reduced growth in the aerial part was given by the aboveground dry mass, which was 43% lower in 2018 and 2020, and 71% lower in 2022 in inoculated maize (**Table 1**). The specific leaf area (SLA, the surface of a unit dry mass of leaves) was unaffected by the fungus in 2020, whereas it was significantly higher in inoculated plants in 2022 (**Table 1**).

The dry mass of the root system was affected by *M. maydis* in a similar way to that of the aboveground mass. The presence of the fungus significantly reduced the mass of the root system by 41%, 46% and 58% in 2018, 2020 and 2022, respectively. The total root length of the adsorbent roots - traditionally those below a 2-mm diameter, McCormack et al. (2015) - was much greater in the larger plants of the 2022 experiment than in the previous ones (**Table 1**). In all three experiments, the fungus-infected plants showed a significant reduction in root length with respect to the control plants. The total length of the root system was reduced by 31%, 40% and 59%, respectively, in 2018, 2020 and 2022. A similar response was also apparent in the total surface of the adsorbing root system, which was reduced by 30%, 38% and 61% in 2018, 2020 and 2022, respectively.

The similar reduction in the growth of the aerial and root parts was generally confirmed by the ratio between the root and aboveground masses, which showed no significant differences between control and inoculated plants (0.61 and 0.64, respectively, in 2018, and 0.88 and 0.82, in 2020, non-dimensional, **Table 1**). On the contrary, the bigger 2022 plants exhibited a significant difference in the root/above ground mass ratio (averages of 0.64 and 0.93 for control and inoculated plants, respectively, **Table 1**).

The ratio between the water-adsorbing and water-transpiring surfaces of the experimental plants was different in different experiments, but was not affected by *M. maydis*. In 2020, this ratio was on average 1.25 in control plants and 1.35 in inoculated ones, whereas in 2022 (when plants had longer root systems) the root/leaves surface ratio was 2.59 and 2.52, respectively, in control and inoculated maize (**Table 1**).

### 3.2 Root morphology: length and diameter

**Figure 2:**
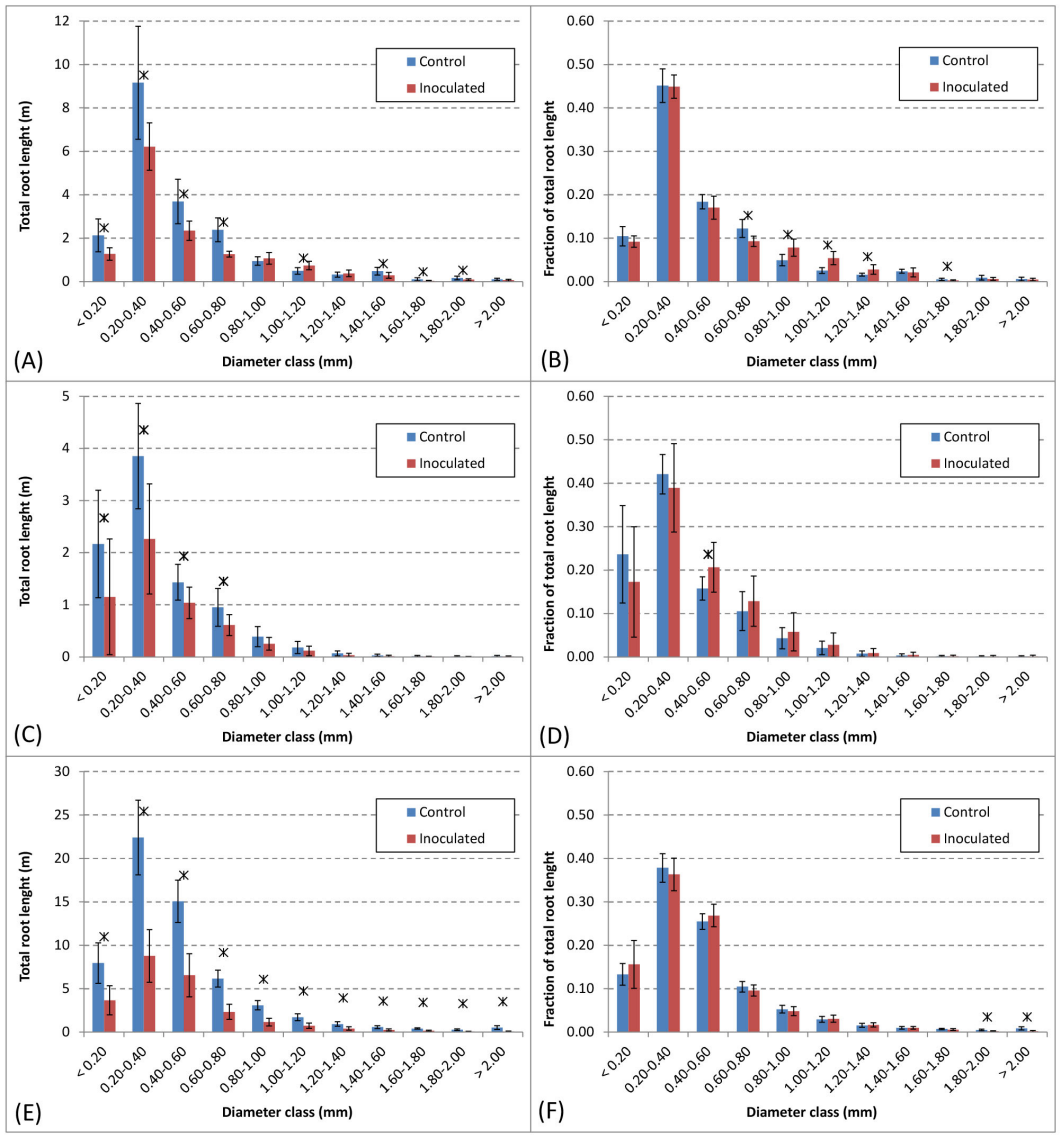
Average distribution of the root length per classes of root diameter in the three experiments: 2018 **(A, B)**, 2020 **(C, D)** and 2022 **(E, F)**. Vertical bars represent standard deviation of the mean of Four replications (plants) for each treatment. Asterisks denote significant differences (Welch T-Test, α=0.05) between the control and the inoculated plants in each class.

The absorbent root systems were analysed to evaluate the hypothesis that the pathogen exerts a distinct influence on the development of roots with varying diameters, which, indirectly, are related to different ages. The results of this analysis are presented in **Figure 2**, illustrating the average absolute root length across diameter categories (**Figures 2A, C, E** for the experiments conducted in 2018, 2020, and 2022, respectively); and, correspondingly, as the average fraction of the total plant root length in **Figures 2B, D, F**.

The root length distribution versus diameter was unimodal in all the experiments, with the 0.20-0.40 class being the one most represented. Even though the root development in the three experiments differed substantially (see also **Table 1**), that of the root length per diameter class was very similar.

In the three experiments, *M. maydis* affected root length significantly in almost all the diameter classes. In the 2020 experiment, which had the smallest root development, the root length from the inoculated treatment was significantly shorter for diameters< 0.8 mm (**Figure 2C**). In the more highly developed root systems of the 2022 experiment (**Figure 2E**), the reduction in root growth due to infection was significant for all the diameter classes of absorbent roots. Nevertheless, the relative impairment caused by the pathogen on root development was mostly always similar in all diameters (**Figures 2B, C, D**). In 2018, some significant differences between treatments in the contribution to the total root lengths were found in the intermediate diameter classes.

### 3.3 Root resistance to water flow of maize infected by *Magnaporthiopsis maydis*

**Figure 3:**
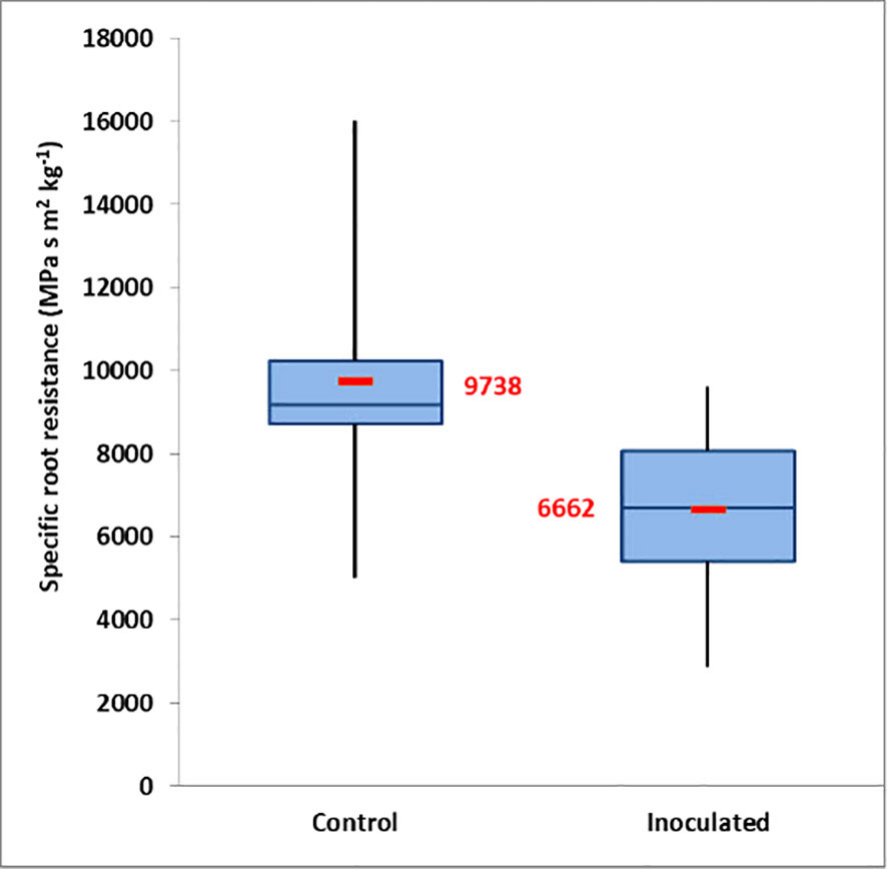
Specific root resistance determined for ten replications (plants) in each control and inoculated treatments in 2022 following Garcia-Tejera et al. (2016). Boxes represent interquartiles, the inner horizontal line is the median, and the whiskers represent the full range of data. Red symbols and labels identify the averages, that were different at P= 0.037 (Welch t-test, n=10).

The measurements of specific root resistance are summarized in the boxplot of **Figure 3**.

The experimental plants showed extreme variability in specific root resistance; in particular, the control ones, whose value ranged approximately from 5000 to 16000 MPa s m^2^ kg^-1^ (**Figure 3**). Nevertheless, no data were found to be at more than 2 standard deviations away from the mean, so, therefore, no outlier was considered. The average specific resistance was 9738 and 6672 MPa s m^2^ kg^-1^ for control and inoculated plants, respectively (**Figure 3**). Despite the high variability, the roots of inoculated plants turned out to have a significantly (*P* = 0.037) lower specific resistance to water flow.

### 3.4 Microbiological and molecular diagnosis of *Magnaporthiopsis maydis* in the inoculated plants

In the three experimental replicates the presence of *M. maydis* was confirmed by mycelial growth development in plates from samples of inoculated plants, but not for control plant plates. The young colonies were white, and they turned to ash-grey with age. They displayed a rhizoid appearance at the edges.

**Figure 4:**
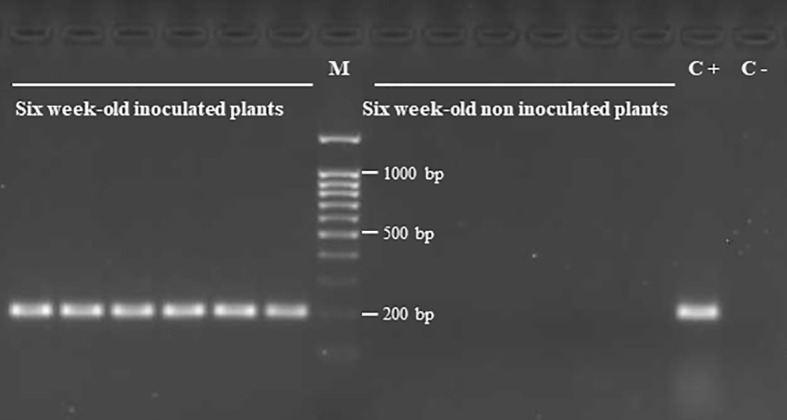
Molecular diagnosis of *Magnaporthiopsis maydis* in low stem tissues of maize plants grown for six weeks under greenhouse conditions. The fungus is identified by the 200-bp amplicon in six individual inoculated plants (lanes to the left of the DNA ladder) as compared to its absence in six non- inoculated plants (lanes to the right of the DNA ladder). M, 100-1,500 bp DNA ladder (Canvax Biotech SL, Cordoba, Spain). Positive (mycelial DNA of Hm01-15 isolate of *M. maydis*) and negative (water) controls of amplification are shown in the last lanes.

The pathogen was also molecularly detected in stem inner tissues of inoculated plants in 2018, 2020 and 2022. **Figure 4** shows the diagnostic 200 bp band in all the DNA samples of inoculated plants from the 2020 replicate. This band was absent in the control plants.

## 4 Discussion

*Magnaporthiopsis maydis* significantly reduced the mass and growth of young maize plants, with detrimental effects on above-ground organs apparent earlier than expected, at around V6-V7 stages. At these early vegetative stages, reductions in stem diameter, height, above-ground biomass and leaf area were quantitatively similar and coherent across experiments (**Table 1**). The pathogen-induced growth reduction was balanced between above-ground organs, yielding anatomically normal but noticeably smaller plants. Similar above-ground biomass and leaf area reductions by *M. maydis* in 80-day plants were reported by Abd el-Rahim et al. (1998). The main symptom, wilting, usually does not appear in young plants (Sunitha et al., 2020). No symptoms of plant wilting were observed in our experiments.

Reduced root mass is a frequent late wilt symptom in adult maize (Ortiz-Bustos et al., 2015; 2016; 2019) and has been recorded in infected young plants (Tej et al., 2018). Our experiments revealed consistent negative impacts of inoculation on root development. In 2018 and 2020, inoculated plants developed root systems of over 40% smaller than controls, escalating to 60% in the larger 2022 plants.

Regarding biomass distribution, in 2018 and 2020, the below-ground to above-ground mass ratio remained unchanged following inoculation, suggesting a proportional reduction in both root and shoot mass while maintaining a balanced structure (**Table 1**). In 2022, when the plants had considerably larger root systems, the pathogen altered this ratio, producing similar root and above-ground masses despite overall growth suppression. This stunted growth in both root and above-ground organs is typical of vascular pathogens like *Verticillium dahliae* infecting tomato, which also cause reduced leaf area, lower photosynthesis rates, and decreased stomatal conductance (Buhtz et al., 2017).

The ratio of the root system total surface area to the foliar surface area indicates plant equilibrium between potential water uptake and transpiration. We tested whether the pathogen might impair root growth or destroy roots, disrupting this balance. Insufficient water uptake could cause water stress symptoms - reduced growth, stomatal closure, cavitation, wilting, and leaf desiccation - resembling *M. maydis* symptoms. However, our experiments demonstrated that the fungus did not alter the root-to-leaf surface area ratio (**Table 1**). Although these findings should be confirmed in the future by measuring hydraulic variables of the whole plant, reduced early-stage growth seems not to be attributable to anatomical imbalance between water source (roots) and sink (leaves) surfaces.

With regard to the average root length, it was substantially lower in inoculated plants, with reductions ranging from 31% (2018) to 59% (2022) (**Table 1**). We investigated whether this effect was associated with specific root classes, potentially altering root diameter distribution; **Figure 2** shows root length distribution per diameter class. All measurements revealed unimodal distributions, with the 0.2-0.4 mm diameter class being the most abundant. Absolute length of each class was lower in inoculated plants (**Figures 2A, C, E**), but relative contribution of each class to total root length remained similar over the years (**Figures 2B, D, F**). The slightly higher contribution of inoculated plants in 0.8-1.4 mm classes observed in 2018 was not confirmed in subsequent experiments. Thus, these results indicate that *M. maydis* reduces the growth of all root diameters equally in young stages.

Unexpectedly, and despite the relatively wide distribution of the root resistance data of our ten replicated measurements, maize root resistance to water flux decreased upon *M. maydis* infection. While vascular fungal pathogens targeting water-conducting xylem vessels from roots upward are well-documented (Yadeta and Thomma, 2013; Thatcher et al., 2016), root physiology alterations in response to vascular fungi are less studied. In vascular pathogen-infected plants, increased water flux resistance hinders transport to upper organs, eventually causing wilting. The pathogen infects through the roots and enters xylem vessels, where it proliferates (Yadeta and Thomma, 2013). However, we observed decreased maize root resistance upon *M. maydis* infection (**Figure 3**), suggesting that the xylem vessel blockage might not be the main effect of the fungus at early-stage plant growth. An alternative hypothesis is that xylem resistance (R_x_) may increase via vessel clogging, while infection simultaneously decreases the (normally much higher) radial resistance (R_r_) - potentially by breaking the Casparian strip and enabling an apoplastic water pathway to the xylem - resulting in similar or decreased overall R_root_ (Equation 2). Fungal disruption of root epidermis and Casparian strip at *Verticillium* spp. entrance sites has been observed (Fradin and Thomma, 2006; Tian and Kong, 2022). However, we did not find the expected R_root_ increase in infected plants, implying that the massive 2022 growth reduction cannot directly result from impaired root water flow. Abd el-Rahim et al. (1998) found significant xylem clogging in *M. maydis*-infected maize stems, and calculated whole-plant R_x_ 10–60 times higher in infected vs. control plants. Their results contrast only superficially with ours, as they studied 80-day-old plants showing wilt symptoms and estimated R_x_ from stem vessel lumen reduction using the Hagen-Poiseuille equation.

**Figure 5:**
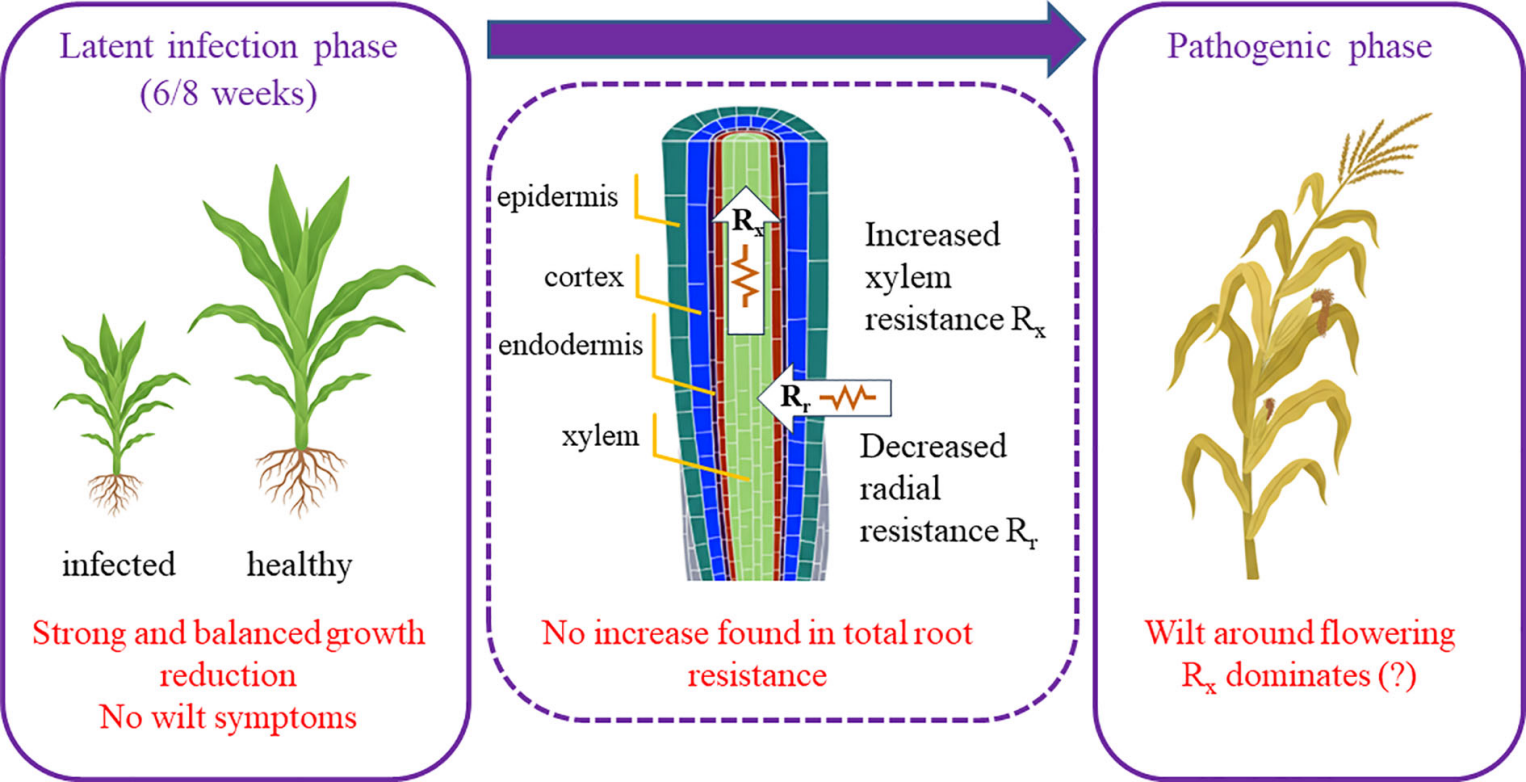
In maize plantlets, a latent phase of *Magnaporthiopsis maydis* growing internally may coincide with a reduced radial root resistance to water flow, masking the vascular effect of the fungus. This transient stage possibly precedes the pathogenic phase, when sudden wilt appears at flowering.

Our results suggest that early growth reduction upon *M. maydis* infection may result not, or not only, from water shortage but from other causes, like toxin transport, as suggested by Sun et al. (2017) for cucumber infected by *Fusarium oxysporum* f. sp. *cucumerinum*, in which the leaf water status remained unaltered before complete wilt. Unaltered water flow in infected roots could also relate to latent fungus-plant interaction. During pathogenesis, some pathogens maintain a latent lifestyle in which plant and pathogen initially co-exist without apparent symptoms, with the disease developing later. For example, *Fusarium proliferatum* (Matsushima) Nirenberg causing vascular garlic wilt can remain latent in bulb tissues long after harvest (Anum et al., 2024). As noted for *Sphaeropsis sapinea* in red pine seedlings (Stanosz et al., 2001), *M. maydis* latency could explain sudden disease development, especially under stress conditions. The strong dependence of late wilt symptoms on environmental factors - temperature, humidity, soil structure, water availability (Abd El-Rahim et al., 1998; Degani et al., 2018; Ortiz-Bustos et al., 2019; Samra et al., 1966) - is consistent with these factors influencing infected plant physiology. Whether latent infection becomes symptomatic or not, may result from the balance of environmental and/or physiological conditions between plant and fungus, as suggested for *Fusarium verticillioides*-maize (Bacon et al., 2008) and *Colletotrichum graminicola*-maize (Sukno et al., 2008) interactions. Moreover, under biotic and abiotic stresses, latent pathogens can fulfil multiplication and trigger infection processes (Brader et al., 2017; Hardoim et al., 2015). As proposed by Mussell (1980), a shift in host physiology (i.e., maize flowering) permits active pathogen development and rapid disease progression. We could hypothesize that *M. maydis* would grow internally without apparently affecting root-to-vascular-system water transport temporarily (latent period), with wilt appearing suddenly later, at around flowering time (pathogen period). However, this statement should be supported in future research by anatomical or histological evidence. Mussel (1980) also notes that latent pathogens maintain a relatively long fungal contact without excessive host damage, which is true for the *M. maydis*-maize pathosystem, and similar to systemic, delayed *Verticillium longisporum* colonization in oilseed rape (Knüfer et al., 2017). Also the establishment of *F. verticillioides* in maize is often asymptomatic during early infection stages, particularly in root and stalk tissues, but progresses to visible symptoms such as rotting, discoloration, and necrosis in fully ripened plants (Gai et al., 2018). The hypothesis that *M. maydis* behaves as a latent pathogen would agree with its previously reported low pathogenicity compared to other maize pathogens like *Fusarium* spp (El-Shafey and Claflin, 1999; Saleh et al., 2003). Indeed, *M. maydis* co-exists very frequently with other faster-growing fungi, particularly *Fusarium* spp., in naturally- infected stem tissues (Degani, 2021; Ortiz-Bustos et al., 2015). This latency hypothesis would explain the unaltered or reduced resistance to water transport from the roots to the vascular system. As graphically presented in **Figure 5**, we hypothesize that despite potential R_x_ increases from vessel clogging, infection may decrease R_r_, resulting in decreased R_root_.

Possible causes of decreased radial resistance in maize upon *M. maydis* infection enabling an apoplastic water pathway to xylem should be explored. Additionally, whether the fungus maintains a latent period, under what circumstances, and to what extent, must be analysed in future research. All these uncertainties, once clarified, will shed light on how to monitor and manage late wilt of maize in a more effective and sustainable manner. In particular, with regard to control tools such as the use of resistant varieties or irrigation management (Degani, 2022; Ortiz-Bustos et al., 2019), early detection of fungal infections—regardless of the presence or absence of symptoms—will make it possible to assess the risk of disease development and support farmers’ decision−making.

In conclusion, *M. maydis* reduces above-ground maize growth earlier than expected, and comparably to root growth reduction. The similarly stunted growth of both root and above-ground organs typifies vascular pathogens. Unexpectedly, at the young developmental stages studied, the fungus reduced the growth of all root diameters equally. Reduced growth symptoms are not attributable to anatomical imbalance between the water source (roots) and sink (leaves) surfaces, nor do they necessarily correlate with an impaired root water flow or fungal xylem vessel blockage.

The relationship of the results of this work about an unaltered water flow in the plantlet with a latent fungal lifestyle should be confirmed in future research.

## Data Availability

The original contributions presented in the study are included in the article material. Further inquiries can be directed to the corresponding author.
